# A Novel Specific Anti-CD73 Antibody Inhibits Triple-Negative Breast Cancer Cell Motility by Regulating Autophagy

**DOI:** 10.3390/ijms20051057

**Published:** 2019-02-28

**Authors:** Zheng Qiao, Xiaoping Li, Nannan Kang, Yue Yang, Chuyuan Chen, Tao Wu, Mingjun Zhao, Yu Liu, Xuemei Ji

**Affiliations:** 1 School of Life Science & Technology, China Pharmaceutical University, Nanjing 210009, China; 14311030143@stu.cpu.edu.cn (Z.Q.); kangnannan@126.com (N.K.); cpuyy4315@126.com (Y.Y.); ccyyao@126.com (C.C.); iioowt0212@163.com (T.W.); zmjun91@126.com (M.Z.); 2 Hainan Institute of drug research, 7 Medicine Valley one road, Haikou 570311, China; yezi-0915@163.com

**Keywords:** anti-CD73 antibody, triple-negative breast cancer, metastasis, autophagy

## Abstract

Increasing researches have focused on cancer metastasis and development. The ectonucleotidase CD73 is one of the most common cell surface enzymes that are involved in immunosuppression. In this study, the recombinant plasmid pET28a-CD73 was constructed and the CD73 protein was overexpressed in *E. coli* as an inclusion body that was then subjected to refolding. The anti-CD73 monoclonal antibody (3F7) was obtained by hybridoma technology. The antibody subtype was identified as IgG2a with an affinity constant of 5.75 nM. This antibody could be applied to immunofluorescence and flow cytometry. The results showed that the CD73 protein was not only located in the cytoplasm but also distributed on the surface of triple-negative breast cancer cells MDA-MB-231 and MDA-MB-468. Moreover, the level of CD73 protein was associated with the survival rate. Although the anti-CD73 antibody was not able to inhibit tumor cell growth, it could enhance the cytotoxic effect of Doxorubicin to triple-negative breast cancer cells. In vitro function assay results indicated that anti-CD73 mAb could inhibit cell migration and invasion in both human triple-negative breast cancer and mouse 4T1 cell lines. In this process, both the LC3I/LC3II ratio and p62 protein levels increased, which indicated that the blockage of CD73 could inhibit cell autophagy, and cell migration and invasion were restored by rapamycin. In vivo, anti-CD73 mAb could significantly inhibit lung metastasis of 4T1 cells in a mouse xenograft model. Taken together, this novel anti-CD73 antibody could be developed as an adjuvant drug for triple-negative breast cancer therapy and can be useful in tumor diagnosis.

## 1. Introduction

Triple-negative breast cancer (TNBC), characterized by the lack of expression of the estrogen receptor (ER), the progesterone receptor (PR), and the human epidermal growth factor receptor 2 (HER2), is a heterogeneous subtype of epithelial breast tumor. It accounts for 15–20% of all breast cancers [1]. A high level of genetic heterogeneity among TNBC has led to poor diagnoses and a lack of an effective targeted therapy, compared with other subtypes [2]. Currently, among the patients with TNBC, the 5-year survival rate is less than 30%; women with TNBC have a tendency for aggressive metastasis to distant vital organs, which leads to shortened survival [3]. Since these metastatic cancers do not respond to some targeted therapies (i.e., Trastuzumab and Lapatinib), adjuvant chemotherapy (i.e., anthracycline) is still a vital therapeutic drug for TNBC. However, chemoresistance remains as an obstacle in clinical settings. Metastasis and drug resistance are urgent problems in the treatment of TNBC.

CD73/Ecto-5′-nucleotidase(eN) is a widely distributed GPI-anchored ectoenzyme that catalyzes the extracellular hydrolysis of 5′-AMP into adenosine [4]. The interaction between CD73-generating adenosine and specific GPCR (G protein-coupled receptor) modifies the tumor immune microenvironment, leading to the growth and metastases of tumors with the homeostatic dysregulation of lymphocytes [5]. It is known that the CD73 protein is highly expressed in various types of cancers, including ovarian cancer, glioma, and breast cancer [6]. In addition, increasing evidence has verified that the level of its expression is associated with drug resistance, tumor metastases, and diagnoses in TNBC patients [7]. 

In some cases, the blockage of the CD73-adenosine pathway obviously improves the antitumor effect of anti-CTLA4 and anti-PD-1 antibody therapies in metastatic 4T1.2 tumor-bearing mice, which is dependent on host IFN-γ and CD8+ T cells while independent of CD4+ T cells [8,9]. CD73 expression could suppress NK and CD8+T cell-mediated immunosuppressive effects and could enhance the resistance to anti-ErbB2 mAb therapy [10]. CD73-specific siRNA reduces the expression and activities of MMP 2 and 9 and improves the efficacy of DC-based cancer immunotherapy in 4T1 bearing mice [11]. Furthermore, CD73 not only impairs tumor immune escape but also maintains endothelial integrity [12]. Consequently, a new biologic adjuvant therapy targeting CD37 might be of great clinical value for cancer patients in the future.

Various studies have demonstrated that anti-CD73 antibody or CD73-interfering RNA has the capacity of delaying breast cancer growth or metastasis [7,13,14]. Although the functions of CD73 protein play an important role in migration and invasion [15], the mechanism by which CD73 is involved in cell motility remains unclear. Autophagy has an impact on metastasis and chemoresistance in TNBC [16,17]. LC3B, a marker of autophagy, is more highly expressed in TNBC than in other subtypes. A high level of LC3B expression predicts a low survival rate of TNBC patients [18]. Furthermore, autophagy inhibition by silencing ATG5 or ATG7 is able to improve chemotherapy sensitivity in high-LC3B triple-negative breast cancer [19], and anti-CD73 mAb therapy also has a similar effect [5]. However, the relationship between CD73 and autophagy has not been elucidated.

In this study, we expressed and obtained the soluble CD73 protein. Then, the clone cell secreting a specific targeting CD73 antibody was screened after the fusion of SP2/0 and splenocytes. The targeting antibody had the ability to inhibit TNBC migration and invasion in vitro, which was related to cell autophagy. Moreover, the novel anti-CD73 antibody could inhibit the lung metastasis of 4T1 cells in vivo. 

## 2. Results

### 2.1. Plasmid Construction and Expression of the CD73 Protein

**Figure 1:**
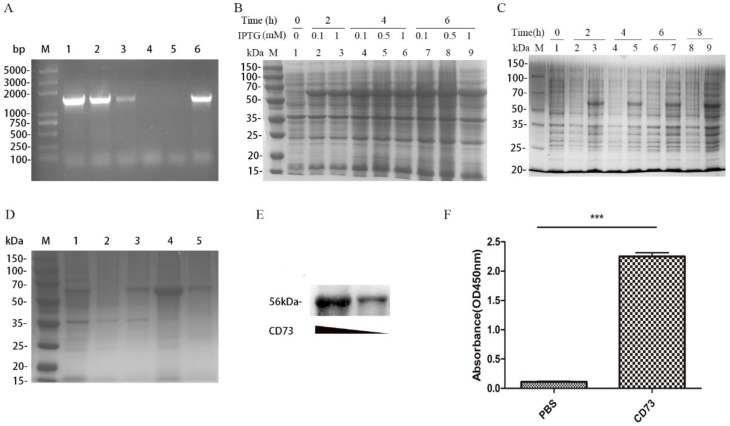
The expression of the CD73 protein: (**A**) the PCR identification of positive clones, where Lane M is the DNA markers and lines 1–6 are the selected clones from the LB plate medium containing kanamycin; (**B**) the SDS-PAGE analysis of the protein expression at different concentrations of IPTG; (**C**) the SDS-PAGE analysis of the expression of the CD73 protein; (**D**) the SDS-PAGE analysis of the refolding of the CD73 protein, where M is the molecular weight markers, line 1 is the BL21 lysate resuspended by PBS, line 2 is the lysate supernatant after ultrasonication, line 3 is the lysate sediments after ultrasonication, line 4 is the inclusion bodies, and line 5 is the soluble CD73 protein after refolding; (**E**) the Western blot of different concentrations of the soluble CD73 antigen; and (**F**) the Enzyme-Linked Immunosorbent Assay (ELISA) analysis of the soluble CD73 protein. Data were shown as means ± SD and analyzed by two tailed *t*-test; *** *p* < 0.001. Data were representative of at least three independent experiments.2.2. Characterization of Anti-CD73 Antibody.

For the expression of the mature CD73 protein, the CD73 gene was amplified by PCR and inserted into a pET-28a (+) vector. Subsequently, the vector was transformed into *E. coli* strain DH5α competent cells and screened on an LB plate supplemented with 50 µg/mL kanamycin. Six positive clones were selected and confirmed by PCR (Figure 1A). Recombinant plasmids (pET28a-CD73) were then transformed into BL21(DE3)-competent cells. An SDS-PAGE analysis showed that a protein (around 56 kDa) was expressed after induction with IPTG at 30 °C and 180 rpm, which was consistent with the expected size of the mature CD73 protein. As shown in Figure 1B, different concentrations of IPTG (0.1, 0.5, and 1 mM) induce the same level of protein expression. In order to identify whether the protein is CD73, Western blot, SDS-PAGE, and Enzyme-Linked Immunosorbent Assay (ELISA) assays was performed. We showed that the protein was expressed in BL21 strains transformed with pET28a-CD73 after the IPTG induction instead of BL21 strains transformed with a pET28a vector (Figure 1C). However, the CD73 protein mainly accumulated as non-native inclusion bodies (Figure 1D). The inclusion bodies of the histidine-tagged CD73 protein were dissolved by a denaturing solution, purified by Ni-NTA agarose, eluted with 50 mM iminazole, and slowly dropped into the refolding solution. Finally, the native form of protein was obtained. ELISA showed the binding between the 1D7 antibody and the CD73 protein (Figure 1F). The western blot results verified the interaction between the 1D7 antibody and the CD73 protein (50 and 200 µg) (Figure 1E), and a high concentration of the CD73 protein could bind more strongly to the 1D7 antibody than a low concentration.

**Figure 2:**
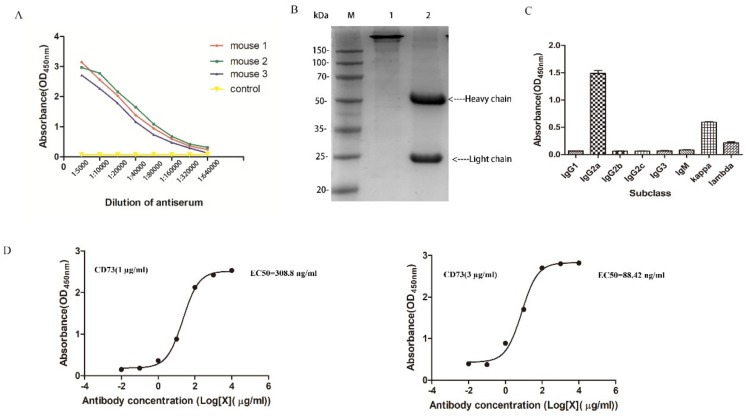
The characterization of CD73 mAb: (**A**) the antibody titer determination; (**B**) the SDS-PAGE analysis of the 3F7 antibody in an oxidizing and reducing loading buffer, where line 1 and line 2 were the 3F7 antibody in oxidation and reduction, respectively; (**C**) the subclass identification of 3F7; and (**D**) the binding curve of 3F7 to the CD73 protein. Left: The concentration of the coated CD73 was 1 μg/mL. Right: The concentration of the coated CD73 was 3 μg/mL. Data are representative of at least three independent experiments and means ± SD were presented.

The soluble CD73 protein was injected into BALB/C mice, and blood samples containing anti-CD73 antibody from orbital vein plexus were used to determine the antibody titer by ELISA. As shown in Figure 2A, the titers of antibody from three of the mice immunized with the CD73 protein were significantly higher than mice with PBS. The results suggested that the CD73 protein was successfully used as an antigen to stimulate the production of anti-CD73 antibodies. After cell fusion, positive clone cells with a high affinity were screened with HAT and HT. Finally, 3F7 clone cells were selected and injected into BALB/C mice. Anti-CD73 antibodies (3F7) were obtained and purified by Protein-A chromatography. SDS-PAGE assays showed that the 3F7 antibody contains a heavy chain around 55 kDa and a light chain around 25 kDa (Figure 2B). ELISA assays showed that the antibody’s heavy chain was IgG2a and the light chain was kappa (Figure 2C). The 3F7 mAb affinity constant (Kaff) was estimated according to Beatty’s method [20]. As shown in Figure 2D, the ELISA experiments showed that the affinity constant of the 3F7 antibody was 5.75 nM and that the antibody could be used for neutralizing the CD73 protein on the cell surface.

### 2.2. Expression of CD73 in Breast Cancer

**Figure 3:**
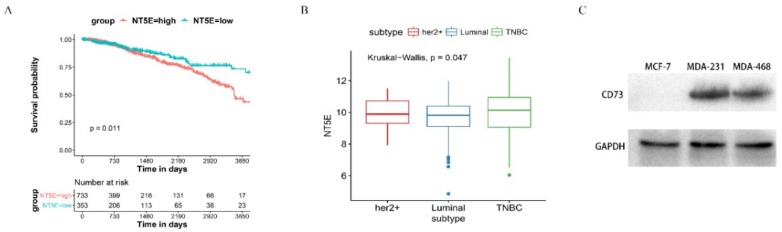
The expression of CD73 and the survival probability in breast cancer: (**A**) a Kaplan–Meier curve was used to detect the overall survival (OS) of CD73 in BC patient, using best separation; (**B**) the expression level of CD73 in Her2+, luminal, and triple-negative breast cancer (TNBC) patients; (**C**) Western blot assay of expression level of CD73. Data are representative of at least three independent experiments and means ± SD were presented.

To validate the potential role of CD73 in TCGA breast cancer data sets, we analyzed the correlation of CD73 expression and the survival probability in 1216 cases. Using the cutoff, a high CD73 expression (733 cases) and a low CD73 expression (353 cases) were classified. The median survival time (MSTs) of NT5E-high patients was 9.5 years (95% CI, 8.94–12.2) for overall survival (OS), while that of NT5E-low patients was 12.4 years (95% CI, 10.81–NR), which had a significant statistic difference (*p* = 0.011). As shown in Figure 3A, the results revealed that a high CD73 expression was associated with a lower survival rate. BC patients containing Her2+ (59 cases), TNBC (113 cases), and Luminal (358 cases) were used to evaluate the level of CD73 expression (Figure 3B). The analysis showed that expression of the protein was upregulated in TNBC samples compared with the other two subtypes (*p* < 0.05). The western blot assay showed that the protein of interest is overexpressed in TNBC cells (MDA-MB-231 and MDA-MB-468) but not detected in MCF-7 cells (Figure 3C).

### 2.3. 3F7 Antibody Specifically Targeted the CD73 In Vitro

**Figure 4:**
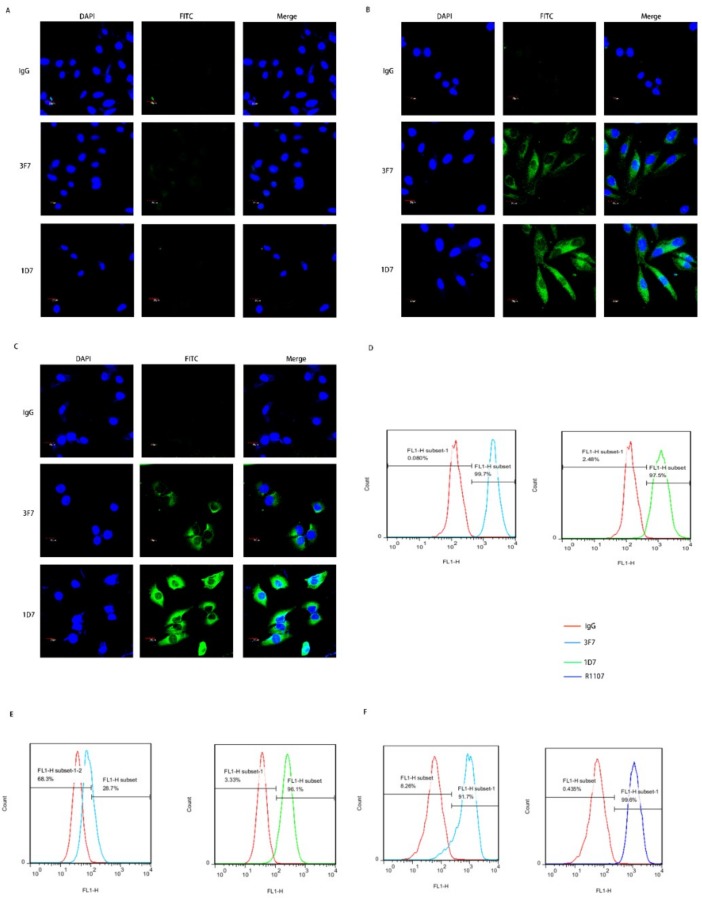
The 3F7 antibody specifically targeted the CD73 in vitro: (**A**–**C**) an indirect immunofluorescence assay showed the binding of antibody with CD73 in MCF-7, MDA-MB-231, and MDA-MB-468 at 630× magnification, respectively, and (**D**–**F**) a flow cytometry analysis of the binding of antibody-CD73 on the surface of MDA-MB-468, MDA-MB-231, and 4T1 cells, respectively; R1107, an antibody targeting mouse or human CD73 protein. Data were representative of at least three independent experiments.

The targeting specificity of the anti-CD73 antibody requires further studies. The purified 3F7 antibody, IgG and 1D7 (positive control), was used as the primary antibody to incubate with breast cancer cells. As mentioned above, the expression of CD73 was not detected in MCF-7 by the Western blot (Figure 3C). The fluorescence signal was also not detected in MCF-7 cells (Figure 4A) but was observed in MDA-MB-231 and MDA-MB-468 cells (Figure 4B,C). Moreover, it showed that a fraction of the CD73 proteins localized in the cytoplasm. CD73 was also detected on the plasma membrane of human breast cancer cells (Figure 4D,E) and mouse 4T1 breast cancer cells (Figure 4F). Our data demonstrated that the 3F7 antibody, like the 1D7 or R1107 antibody, could target CD73.

### 2.4. 3F7 Antibody Enhanced Cytotoxic Effects of DOX

**Figure 5:**
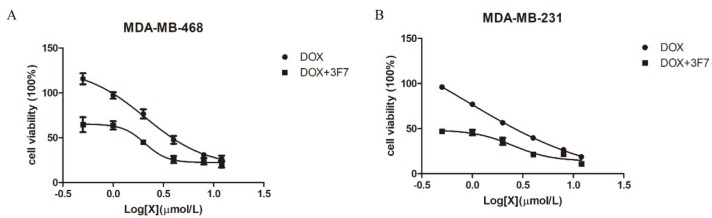
The 3F7 antibody enhanced the cytotoxic effects of Doxorubicin (DOX): (**A**) the proliferation of MDA-MB-468 cells in the presence of DOX with or without 3F7 mAb and (**B**) the proliferation of MDA-MB-231 cells in the presence of DOX with or without 3F7 mAb. Data were representative of at least three independent experiments and means ± SD were presented.

TNBC patients do not respond to targeted therapy and endocrine therapy. Currently, chemotherapy and radiotherapy are the primary treatments for TNBC, and Doxorubicin (DOX) is a commonly used antineoplastic drug in clinical settings. However, chemoresistance leads to a lower survival probability of patients with TNBC, and some patients have a tumor recurrence and DOX resistance. To further study the function of our antibody, we investigated the role of the 3F7 antibody in TNBC cell proliferation. As shown in Figure 5A,B, we confirmed that the 3F7 antibody could not decrease the viability of MDA-MB-231 and MDA-MB-468 cells (data not shown). However, DOX could damage MDA-MB-231 (IC50 = 2.939 µM) and MDA-MB-468 cells (IC50 = 4.635 µM), and TNBC cells were more sensitive to MDA-MB-231 (IC50 = 0.538 µM) and MDA-MB-468 cells (IC50 = 1.463 µM) after DOX treatment when the 3F7 antibody was added into the cell medium.

### 2.5. Anti-CD73 Antibody Inhibits Invasion and Migration of TNBC In Vitro

**Figure 6:**
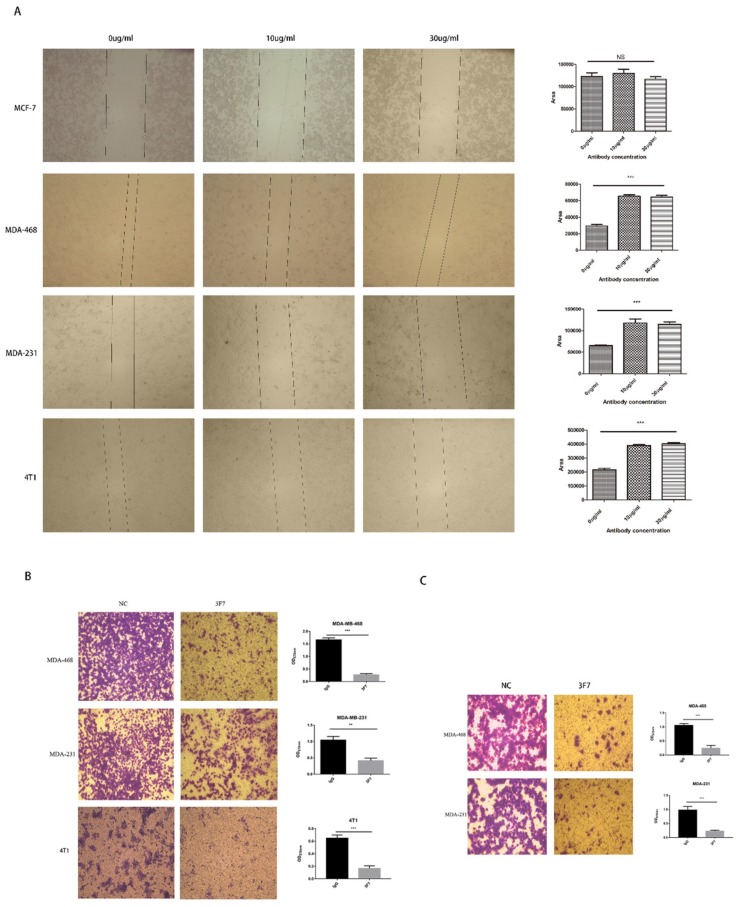
The analysis of the migration and invasion of cells treated with the anti-CD73 antibody: (**A**) the wound healing assay showed that different concentrations of 3F7 mAb inhibited the cell migration (40×) and (**B**,**C**) the Transwell assays showed that 3F7 (10 µg/mL) inhibited the migration and invasion of cells (40×), respectively. Data were shown as means ± SD and analyzed by two tailed *t*-test; NS, not significant; ** *p* < 0.01; *** *p* < 0.001. Data were representative of at least three independent experiments.

For the determination of the antibody bioactivity, a wound healing assay and a Transwell assay were conducted. Because of the overexpression of CD73 in TNBC but not in other breast cancer subtypes, MDA-MB-231, MDA-MB-468, and 4T1 cells were selected for the subsequent experiments. The wound healing assay showed that cell migration was more significantly inhibited after 3F7 antibody treatment compared with IgG treatment (Figure 6A), but inhibition rates were the same at different concentrations of antibody (10 and 30 µg/mL). To further assess the migration of TNBC, 10 µg/mL 3F7 antibody was placed in Transwell inserts with cells, which led to a significant decrease in the number of migrated cells that crawled through the insert membrane (Figure 6B). In invasion experiments, the cell numbers that invaded though the matrigel and membrane to the lower compartment were measured. The results indicated that treatment with the 3F7 antibody was responsible for diminishing their invasive capacity (Figure 6C).

### 2.6. Anti-CD73 Antibody 3F7 Repressed 4T1 Cell Lung Metastasis

**Figure 7:**
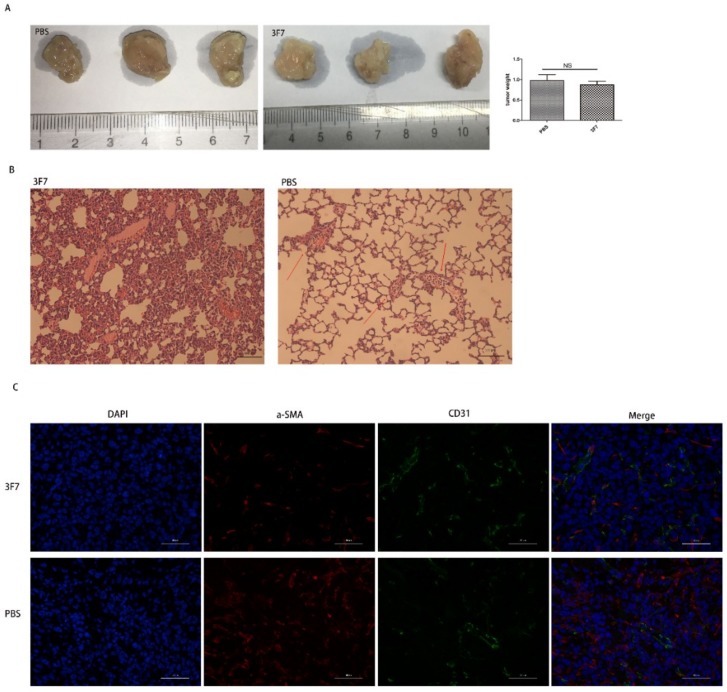
The 3F7 antibody repressed the 4T1 lung metastasis in vivo: (**A**) the tumor weight after treatment with 3F7 or PBS; (**B**) the mouse 4T1 breast cancer lung metastasis (200×); and (**C**) the indirect immunofluorescent double staining with the α-SMA antibody and CD31 antibody (400×). The data are shown as means ± SD (*n* ≥ 3) and analyzed by *t*-test; NS, not significant.

The above results also showed that the migration of metastatic 4T1 cells was repressed by 3F7 mAb (Figure 6A and B), suggesting that 3F7 mAb could bind to the mouse CD73 protein. To investigate the antitumor ability of the anti-CD73 antibody in mice, 4T1 cells were inoculated subcutaneously into BALB/C mice. After treatment with 3F7 (10 mg/kg), mice were sacrificed and the tumors were weighed, and the results showed that 3F7 mAb monotherapy had no significant effect compared with PBS treatment on tumor size (Figure 7A). Hematoxylin and eosin (H&E) staining showed that 3F7 treatment significantly inhibited the 4T1 lung metastasis (Figure 7B). In the wound healing assay, we also demonstrated that 3F7 was capable of suppressing the migration of hCEMC/D3 endothelial cells (data not shown). There are some reports that CD73 could modulate the functions of endothelial cells [21]. Therefore, we postulated that the CD73 protein played a role in vascular normalization. Pericytes and smooth muscle cells are required for the stabilization of maturing blood vessels; we chose α-SMA as a marker for mature pericytes and CD31 as a marker for endothelial cells. CD31 staining suggested that the vascular architecture of both 3F7 and PBS-treated groups were chaotic and disordered. Double staining for CD31 and α-SMA showed no more pericytes covered the blood vessels (α-SMA) in mice treated with 3F7 when compared with PBS, indicating similar immature tumor vessels (Figure 7C), a sign of vessel disorganization. Images showed that α-SMA pericytes had no more orderly attachment to tumor vessels in treatment with the 3F7 antibody (Figure 7C).

### 2.7. Anti-CD73 Antibody 3F7 Inhibited the Migration and Invasion by Modulating Autophagy

**Figure 8:**
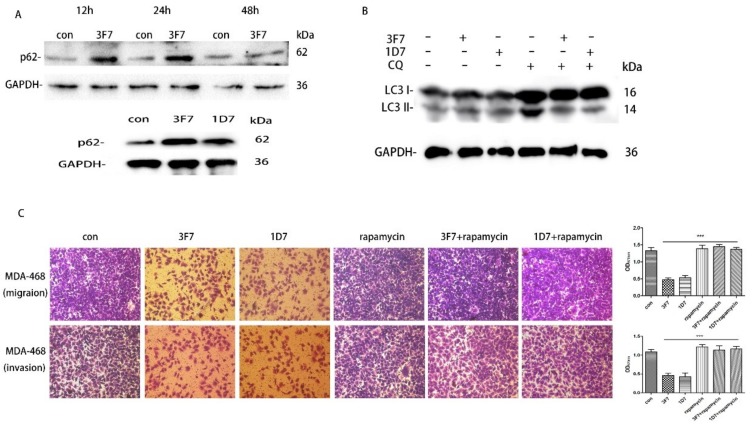
Autophagy participated in the inhibition of cell migration and invasion after treatment with 3F7: (**A**) MDA-MB-468 cells were treated with 3F7 (10 μg/mL) or 1D7 (down) for 12, 24, and 48 h. p62 and GAPDH were detected by a Western blot analysis; (**B**) MDA-MB-468 cells were treated with 3F7 (10 μg/mL) or 1D7 for 24 h, and LC3 and GAPDH were detected by a Western blot analysis; and (**C**) Rapamycin restored the inhibition of cell invasion (up) and migration (down) after treatment with 3F7 or 1D7 (100×). Rapamycin: autophagy stimulator. Data are shown as means ± SD and analyzed by *t*-test; NS, not significant; *** *p* < 0.001. Data are representative of at least three independent experiments.

It is established that autophagy participates in tumor metastasis and chemoresistance [17,19]. LC3, an autophagic marker protein, is also associated with progression and poor survival in TNBC [18]. Therefore, we investigated the relationship between CD73 and autophagy. The conversion of LC3-II/LC3-I indicates a reduced rate of autophagy, while an increase in p62 level is considered as an indicator of suppressed autophagy since p62 is a substrate of the autophagic-lysosomal degradation pathway. As shown in Figure 8A, we detected the expressed level of p62 by a Western blot assay at 12, 24, and 48 h. The assay showed that p62 proteins significantly increased when MDA-MB-468 cells were treated with 3F7 for 12 and 24 h. However, there was no significant difference in p62 expression at 48 h. Furthermore, p62 proteins also significantly increased in treatment with the 1D7 antibody at 24 h. We also found that the ratio of LC3-II/LC3-I significantly decreased with treatment of 3F7 or 1D7 antibody (Figure 8B), which demonstrated that autophagic activity was inhibited by the antibody in MDA-MB-468. Next, Transwell migration and invasion assays showed that inhibited cell migration and invasion due to treatment with 3F7 or 1D7 was restored by rapamycin, an mTOR inhibitor that can induce autophagy (Figure 8C).

## 3. Discussion

Invasive metastasis is the leading challenge in the treatment of TNBC patients. These metastatic patients have a poor prognosis and shorter median time to relapse and death [22]. In this study, we analyzed the data from TCGA and found that a high expression of CD73 in breast cancer was significantly correlated with a poor overall survival (OS) time, which was in accordance with a previous report [23]. In view of the analysis of these data, targeting the CD73 protein could improve clinical outcomes in TNBC. Importantly, thus far, there is currently no standard therapy for TNBC patients.

For the preparation of specific anti-CD73 antibodies, the soluble CD73 protein was expressed using multiple approaches. A yeast shuttle vector was used to construct the recombinant plasmid pPICZα(A)-CD73. Subsequently, the SacI-linearized plasmid was transformed into the *P. pastoris* GS115 strain. Positive clones were selected to detect the level of CD73 expression. Unfortunately, the Western blot assay showed that these clones were unable to express the protein of interest. In some cases, proteins overexpressed in prokaryotic cells are unable to fold properly and, thus, form non-native proteins. We made attempts to express the small ubiquitin-related modifier (SUMO)-tag CD73 protein. Although there has been some reported success of improving the protein solubility by fusing with solubility tags including glutathione-S-transferase (GST), maltose binding protein (MBP), and SUMO [24], the SUMO-CD73 fusion protein remained insoluble in *E. coli*. Finally, the refolding of the CD73 protein was considered and performed. In the process, zinc chloride was added to facilitate the renaturation of the protein of interest, which was possibly due to the enzyme structure of CD73. The stability and activity of CD73 may be zinc-dependent [25].

The antibody-mediated immune checkpoint blocker approved for cancer treatment has been closely investigated. The combination of the anti-CD73 antibody with the novel immune checkpoint antibody (CTLA-4 antibody, PD-1 antibody) as well as with conventional anticancer agents could improve the efficacy of therapy [8]. In this study, we used the CD73 protein as an antigen to immune BALB/C mice. Cell fusion was performed when antibody titers against CD73 in serum reached 1:320000 after the 3rd immunization. The specific binding of the IgG2a antibody subtype (3F7) with the protein has been verified in the above results. Meanwhile, it was demonstrated that CD73 was expressed both intracellularly and on the surface of MDA-MB-231 and MDA-MB-468 cells. A similar result was reported in COS7, lymphocytes, and endothelial cells [26,27] but not reported in TNBC. A shuttle between intra- and extracellular 5′-nucleotidase pools may play a part in biological functions that remains unclear. Therefore, there may be different roles in targeting the anti-CD73 antibody and siRNA in anticancer therapy. Nevertheless, antibody therapy might lead to better responses from patients, since the anti-CD73 antibody could trigger immune activation and shape local tumor microenvironments [28]. In addition, antibodies have a higher specificity and a longer half-life compared with siRNA or small-molecule inhibitors. In the wound healing assay, we also found that 3F7 was capable of suppressing the migration of hCEMC/D3 endothelial cells. Vascular abnormalities could contribute to tumor cell metastasis Therefore, we investigated whether anti-CD73 antibody therapy improved vascular integrity. Pericytes and smooth muscle cells are required for the stabilization of maturing blood vessels, but pericytes did not exhibit more orderly attachment to the tumor vessels after treatment with anti-CD73 antibody.

We found that the binding of the 3F7 antibody to the target protein is lower than the 1D7 antibody on the cells (MDA-MB-468 and MDA-MB-231), one of the reasons is that the 3F7 antibody is an IgG2a subtype and that 1D7 is an IgG1 subtype. A fluorescein isothiocyanate (FITC)-conjugated anti-mouse antibody might bind with a higher affinity to IgG1 than IgG2a, but the results showed that the 3F7 antibody can bind to the CD73 protein. Our data demonstrated that the 3F7 mAb significantly inhibited migration and invasion in triple-negative breast cancer cells. Moreover, 3F7 mAb enhanced the antineoplastic activity of DOX, and MDA-MB-468 and MDA-MB-231 cells were more sensitive to DOX when 3F7 mAb was added into the cell medium. Although 3F7 mAb was shown to protect patients from lung metastasis of breast cancer, tumor growth was not delayed in tumor-bearing mice, which is in agreement with Terp’s findings [29]. However, some other studies have shown different results [14,30]. There might be three reasons for this. Firstly, owing to antibody subtype, the suppression of solid tumors required both enzyme inhibition and activation of Fc receptors [31,32], but our IgG2a subtype failed to strongly activate the immune cells. Secondly, the mouse xenograft model might not more closely simulate the tumor microenvironment than the in situ tumor model, and the anti-CD73 antibody failed to effectively stimulate the immunocyte response. At last, the different CD73 epitope of the antibody could have produced different results, and the mechanism of the interaction between the CD73 protein and its ligand deserves more attention and investigation. The interaction between CD73 and tenascin C (TnC) phosphorylates focal adhesions kinase (FAK), which controls focal adhesions (FA) turnover and, subsequently, mediates cell–cell adhesion [15]. Autophagy also promoted FA accumulation and tumor cell motility [33]. Our studies suggested that the anti-CD73 antibody could mediate autophagy to inhibit cell motility. We searched the candidates that can interact with CD73 from the BioGRID Database (https://thebiogrid.org). It seemed that mTOR was likely to bind with CD73. It has been reported that phosphorylated Akt activates the mTOR complex to inhibit autophagy in nutrient-rich conditions [34]. We need to further study the relationship between CD73, mTOR, and autophagy, which may contribute to understanding why the anti-CD73 antibody enhances chemosensitivity and inhibits tumor metastases. We need to further understand the biological role of the CD73 protein in tumor cells, and the autophagy inhibitor might become a therapeutic drug for TNBC. In summary, the anti-CD73 antibody might be of great clinical value as an adjuvant therapeutic option for triple-negative breast cancer in the future.

## 4. Materials and Methods

### 4.1. Materials

Host strain *E. coli* DH5α, BL21(DE3), and pET28a (+) were purchased from Invitrogen. T4 DNA ligase and restriction endonucleases EcoRI and XhoI were purchased from Takara. Anti-CD73 antibody (1D7) was purchased from Abcam (clone 1D7, catalog no. ab91086, Species reactivity: human); anti-CD73 antibody (R1107) was purchased from Huabio (China, product code: R1107-6, species reactivity: human, mouse), and they were used as a positive control antibody. The full-length DNA clone of Homo sapiens CD73 (NM_002526, NT5E gene) was obtained from Sino Biological (Beijing, China); NI-NTA and protein A-Sepharose were purchased from GE Healthcare; and anti-LC3B and p62 antibody were from Cell Signaling Technology (CST). MTT (3-(4,5-dimethylthiazol-2-yl)-2,5-diphenyltetrazolium bromide) and Doxorubicin (DOX) were from Sigma-Aldrich (USA). Rapamycin and Chloroquine (CQ) was from MedChemExpress (MCE, Monmouth Junction, NJ, USA).

### 4.2. Cell Culture

Triple-negative breast cancer (human MDA-MB-231, MDA-MB-468, and mouse 4T1 cells) and MCF-7 cancer cell lines were obtained from the American Type Culture Collection (ATCC, Manassas, VA, USA), and mouse myeloma cell line SP2/0 was from Type Culture Collection of Chinese Academy of Sciences (Shanghai, China). MDA-MB-231 and 4T1 were cultured in a L15 and RPMI- 1640 medium (containing 10% FBS (Tianhang Biotechnology, Huzhou, China) and antibiotics (Gibco, Waltham, MA, USA), respectively. MCF-7, MDA-MB-468, and SP2/0 were grown in Dulbecco’s modified Eagle’s medium (DMEM). All cells were maintained at 37 °C and 5% CO_2_.

### 4.3. Construction of pET28a-CD73/BL21, Expression of CD73, and Refolding of the Inclusion Body

A DNA fragment encoding CD73 (27–549 aa) was amplified by PCR with two oligonucleotides used as primers and then inserted in the pET28a (+) vector (forward primer: CGGAATTCTGG-GAGCTTACGATTTTGCAC; reverse primer: CCGCTCGAGGGAAAACTTGATCCGAC-CTT). After digestion, ligation, and sequencing, the recombinant plasmid pET28a-CD73 was transformed into *E. coli* BL21 and cultured overnight at 37 °C in 4 mL of a LB medium (50 µg/mL kanamycin). Protein expression was induced with 0.1 mM IPTG at 25 °C. After 10 h, cells were harvested by centrifugation, resuspended in PBS, and disrupted by sonication. Next, the inclusion bodies were isolated by centrifugation and resuspended in a denaturing solution containing 8 M urea, 50 mM Tris-HCl, and 200 mM NaCl (pH 9.0). Then, the protein was purified using NI-NTA and slowly diluted into a refolding buffer at 4 °C (50 mM Tris-HCl, 200 mM NaCl, 0.5 M L-arginine, 10% glycerol, 4 mM reduced glutathione, 1.8 M urea, 30 µM ZnCl2, 0.4 mM oxidized glutathione, and pH 9.0). After 24 h, the refolding protein solution was dialyzed twice and stored at −20 °C.

### 4.4. Enzyme-Linked Immunosorbent Assay (ELISA)

The binding affinity analysis was performed by ELISA in 96-well plates. The CD73 protein (1 or 3 µg per well) was coated in a coating buffer (pH 9.6, 0.1 M bicarbonate) and washed in PBST (PBS + 0.1%Tween). A blocking buffer (3% BSA in PBST) was used to block nonspecific binding sites. The anti-CD73 antibody (1D7 or 3F7) or cell medium was added to each well and then incubated for 45 min with horseradish peroxidase (HRP)-conjugated anti-mouse antibody (1:5000). TMB substrates (Beyotime, Shanghai, China) were added into each well. After 30 min, 2 M sulfuric acid was added to stop the reaction and A_450nm_ was measured using a microplate reader (Bio-Rad, USA).

### 4.5. Cell Invasion and Migration Assay

MDA-MB-468 cells (4 × 10^4^) or MDA-MB-231 cells (2 × 10^5^) in a medium with 1% FBS and 10µg/mL anti-CD73 antibody (1D7 or 3F7) with or without rapamycin (20 nM) were plated on the upper chamber of Transwell Chambers (8-µm pore size, Corning, NY, USA) with Matrigel for the invasion assay. The cell medium (containing 10% FBS) was added into the lower chamber. After 22 h at 37 °C, cells on the outside of the Transwell filter were fixed in 4% paraformaldehyde and stained with 0.1% crystal violet dye. After being washed by PBS, membranes were air-dried and dipped in 33% acetic acid for 15 min. The wound healing assay was described by Jiang [35].

### 4.6. Anti-CD73 mAb Generation

All mice protocols were approved by the Animal Care and Use Committee of China Pharmaceutical University (Nanjing, China). Eight-week-old BALB/c female mice were purchased from the Comparative Medicine Center of Yangzhou University and immunized subcutaneously with 20–50 μg of CD73 protein in Freund’s complete adjuvant or non-complete adjuvant (Sigma, Ronkonkoma, NY, USA) every 3 weeks. Blood samples were collected from the orbital vein and immediately transferred into centrifuge tubes for centrifugation in the first week after the third immunization. Antibody titers were detected by ELISA. Splenocytes were collected and fused with SP2/0 after the sacrifice of mice by cervical dislocation. Positive clones expressing the anti-CD73 antibody were screened by ELISA and injected intraperitoneally into the BALB/c female mouse to generate antibodies. The antibodies were purified by Protein A-Sepharose.

### 4.7. Immunofluorescence and Flow Cytometry

Breast cancer cells were fixed in 4% paraformaldehyde and then blocked in PBS (3% bovine serum albumin). Cells were stained with the anti-CD73 antibody (1D7, 3F7 and R1107) or IgG at a dilution of 1:100 and then washed and incubated with a FITC-conjugated anti-mouse antibody (1:100, Abcam, UK) in PBS for 45 min. 4′,6-diamidino-2-phenylindole (DAPI) was used for nuclear staining. A confocal microscope (Olympus, Tokyo, Japan) was used for imaging.

Flow cytometry was used to analyze the antibody binding specificity. The cells were trypsinized and washed twice with PBS on ice. After centrifugation for 5 min at 0.4 g, the cell pellets were subsequently resuspended in a blocking buffer (PBS with 5% FBS and 3% BSA). The cells were stained with the anti-CD73 antibody or IgG (1:100) for 1 h at 4 °C. Next, the cells were washed with PBS and incubated with a secondary antibody (1:100) for 40 min. The flow cytometry was performed with a FACSCalibur flow cytometer (BD, San Jose, CA, USA).

### 4.8. Experimental Tumor Metastasis

Eight-week-old female BALB/c mice (n = 6/group) were injected subcutaneously with 4T1 cells (1 × 10^6^) into the right-front dorsum. The animal body weight and tumor size were measured and recorded. After one week, mice were treated by an intraperitoneal injection of PBS or 3F7 antibody (10 mg/kg) every 3 days. After 6 injections, the mice were sacrificed and the tumor tissues were weighed. Then, the tumor and lung tissues were fixed in 4% formaldehyde for further analysis.

### 4.9. Immunohistochemistry and Fluorescence Staining

The protocol was described in our previous report [36]. Briefly, tumor and lung samples were fixed in 4% formaldehyde. After embedding, dewaxing, and rehydration, lung tissue sections were subjected to routine hematoxylin and eosin (H&E) staining. The tumor tissues were incubated with the primary mouse CD31 antibody (1:50, CST, USA) and the rabbit α-smooth muscle actin (α-SMA) antibody (1:50, CST, USA). Subsequently, the cells were stained by anti-mouse Alexa-Fluorl-488 or anti-rabbit Alexa-Fluorl-546 (1:200, Invitrogen, USA). Finally, the cells were stained with DAPI and observed with a fluorescence microscope (Zeiss, Germany).

### 4.10. Cell Proliferation Assay

MDA-MB-468 Cells (7 × 10^3^) or MDA-MB-231 cells (9 × 10^3^) in a medium with 10% FBS were plated in 96-well plates (Thermo Fisher, Waltham, MA, USA), After 22 h, these media were removed, and the medium with 10% FBS and the 3F7 antibody (10 µg/mL) with Doxorubicin (final concentration of 0.5, 1, 2, 4, 8, and 12 µM) was added into plates and incubated for 48 h. Then, 20 µL of MTT (5 mg/mL) were added into the medium. After incubation at 37 °C for 4 h, the cell medium was removed and 150 µL of DMSO was added into each well. Crystallization is fully dissolved in 10 min. Finally, A_570nm_ was measured using a microplate reader.

### 4.11. Western Blot and SDS-PAGE

MDA-MB-468 cells were centrifuged for 3 min at 500 g after trypsinization, and the medium was removed. Next, cells were resuspended and washed by PBS, and then these cells in a medium with 10% FBS were plated in 6-well plates, grown to approximately 80% confluent at 24 h. Rapamycin (20 nM) and CQ (50 μM) were added into 6-well plates. After 24 h or 48 h, the cells were harvested and shaken vigorously with RIPA (Beyotime) and 1 mM phenylmethanesulfonyl fluoride (PMSF) (Beyotime); the proteins were analyzed using a 15% or 12% separation gel. For SDS-PAGE, the CD73 protein was visualized by Coomassie Brilliant Blue. For the Western blot analysis, the total proteins were transferred to an isopropyl β-D-thiogalactoside (PVDF) membrane. After incubation with the anti-LC3B antibody (1:1000), anti-p62 antibody (1:1000), or anti-CD73 antibody (1D7,1:1000), the proteins on the membrane were washed by TBSTand incubated with the HRP-conjugated antibody for 45 min. The proteins were detected using ChemiDoc XRS+ imaging systems (Bio-Rad, Hercules, CA, USA).

### 4.12. TCGA Analysis

The gene expression data and clinical data regarding breast cancers were downloaded from TCGA (http://cancergenome.nih.gov/). Kaplan–Meier plots were used to estimate the prognostic value of CD73 in breast cancer. All the data were analyzed using the R software package.

### 4.13. Statistical Analysis

The data are represented as means ±SD of at least three independent experiments. Student’s unpaired *t*-test was performed. Differences were considered significant when the *p*-value was <0.05.

## References

[1] Lehmann B.D., Bauer J.A., Chen X., Sanders M.E., Chakravarthy A.B., Shyr Y., Pietenpol J.A. (2011). Identification of human triple-negative breast cancer subtypes and preclinical models for selection of targeted therapies. J. Clin. Investig..

[2] Gluz O., Liedtke C., Gottschalk N., Pusztai L., Nitz U., Harbeck N. (2009). Triple-negative breast cancer--current status and future directions. Ann. Oncol..

[3] Anders C.K., Carey L.A. (2009). Biology, metastatic patterns, and treatment of patients with triple-negative breast cancer. Clin. Breast Cancer.

[4] Burnstock G., Di Virgilio F. (2013). Purinergic signalling and cancer. Purinergic Signal.

[5] Allard B., Beavis P.A., Darcy P.K., Stagg J. (2016). Immunosuppressive activities of adenosine in cancer. Curr. Opin. Pharmacol..

[6] Antonioli L., Yegutkin G.G., Pacher P., Blandizzi C., Hasko G. (2016). Anti-CD73 in cancer immunotherapy: Awakening new opportunities. Trends Cancer.

[7] Loi S., Pommey S., Haibe-Kains B., Beavis P.A., Darcy P.K., Smyth M.J., Stagg J. (2013). CD73 promotes anthracycline resistance and poor prognosis in triple negative breast cancer. Proc. Natl. Acad. Sci. USA.

[8] Allard B., Pommey S., Smyth M.J., Stagg J. (2013). Targeting CD73 enhances the antitumor activity of anti-PD-1 and anti-CTLA-4 mAbs. Clin. Cancer Res..

[9] Mittal D., Young A., Stannard K., Yong M., Teng M.W., Allard B., Stagg J., Smyth M.J. (2014). Antimetastatic effects of blocking PD-1 and the adenosine A2A receptor. Cancer Res..

[10] Turcotte M., Allard D., Mittal D., Bareche Y., Buisseret L., Jose V., Pommey S., Delisle V., Loi S., Joensuu H. (2017). CD73 Promotes Resistance to HER2/ErbB2 Antibody Therapy. Cancer Res..

[11] Jadidi-Niaragh F., Atyabi F., Rastegari A., Kheshtchin N., Arab S., Hassannia H., Ajami M., Mirsanei Z., Habibi S., Masoumi F. (2017). CD73 specific siRNA loaded chitosan lactate nanoparticles potentiate the antitumor effect of a dendritic cell vaccine in 4T1 breast cancer bearing mice. J. Control. Release.

[12] Jalkanen S., Salmi M. (2008). VAP-1 and CD73, endothelial cell surface enzymes in leukocyte extravasation. Arterioscler. Thromb. Vasc. Biol..

[13] Zhi X., Chen S., Zhou P., Shao Z., Wang L., Ou Z., Yin L. (2007). RNA interference of ecto-5′-nucleotidase (CD73) inhibits human breast cancer cell growth and invasion. Clin. Exp. Metastasis.

[14] Stagg J., Divisekera U., McLaughlin N., Sharkey J., Pommey S., Denoyer D., Dwyer K.M., Smyth M.J. (2010). Anti-CD73 antibody therapy inhibits breast tumor growth and metastasis. Proc. Natl. Acad. Sci. USA.

[15] Sadej R., Skladanowski A.C. (2012). Dual, enzymatic and non-enzymatic, function of ecto-5′-nucleotidase (eN, CD73) in migration and invasion of A375 melanoma cells. Acta Biochim. Pol..

[16] Aydinlik S., Erkisa M., Cevatemre B., Sarimahmut M., Dere E., Ari F., Ulukaya E. (2017). Enhanced cytotoxic activity of doxorubicin through the inhibition of autophagy in triple negative breast cancer cell line. Biochim. Biophys. Acta.

[17] Mowers E.E., Sharifi M.N., Macleod K.F. (2017). Autophagy in cancer metastasis. Oncogene.

[18] Zhao H., Yang M., Zhao J., Wang J., Zhang Y., Zhang Q. (2013). High expression of LC3B is associated with progression and poor outcome in triple-negative breast cancer. Med. Oncol..

[19] Lefort S., Joffre C., Kieffer Y., Givel A.M., Bourachot B., Zago G., Bieche I., Dubois T., Meseure D., Vincent-Salomon A. (2014). Inhibition of autophagy as a new means of improving chemotherapy efficiency in high-LC3B triple-negative breast cancers. Autophagy.

[20] Beatty J.D., Beatty B.G., Vlahos W.G. (1987). Measurement of monoclonal antibody affinity by non-competitive enzyme immunoassay. J. Immunol. Methods.

[21] Zukowska P., Kutryb-Zajac B., Toczek M., Smolenski R.T., Slominska E.M. (2015). The role of ecto-5′-nucleotidase in endothelial dysfunction and vascular pathologies. Pharmacol. Rep..

[22] Dent R., Trudeau M., Pritchard K.I., Hanna W.M., Kahn H.K., Sawka C.A., Lickley L.A., Rawlinson E., Sun P., Narod S.A. (2007). Triple-negative breast cancer: Clinical features and patterns of recurrence. Clin. Cancer Res..

[23] Jiang T., Xu X., Qiao M., Li X., Zhao C., Zhou F., Gao G., Wu F., Chen X., Su C. (2018). Comprehensive evaluation of NT5E/CD73 expression and its prognostic significance in distinct types of cancers. BMC Cancer.

[24] Malhotra A. (2009). Tagging for Protein Expression. Method Enzymol..

[25] Beck F.W.J., Kaplan J., Fine N., Handschu W., Prasad A.S. (1997). Decreased expression of CD73 (ecto-5′-nucleotidase) in the CD8+ subset is associated with zinc deficiency in human patients. J. Lab. Clin. Med..

[26] Airas L., Niemela J., Salmi M., Puurunen T., Smith D.J., Jalkanen S. (1997). Differential regulation and function of CD73, a glycosyl-phosphatidylinositol-linked 70-kD adhesion molecule, on lymphocytes and endothelial cells. J. Cell Biol..

[27] Fausther M., Lavoie E.G., Goree J.R., Baldini G., Dranoff J.A. (2015). NT5E Mutations That Cause Human Disease Are Associated with Intracellular Mistrafficking of NT5E Protein. PLoS ONE.

[28] Beavis P.A., Stagg J., Darcy P.K., Smyth M.J. (2012). CD73: A potent suppressor of antitumor immune responses. Trends Immunol..

[29] Terp M.G., Olesen K.A., Arnspang E.C., Lund R.R., Lagerholm B.C., Ditzel H.J., Leth-Larsen R. (2013). Anti-human CD73 monoclonal antibody inhibits metastasis formation in human breast cancer by inducing clustering and internalization of CD73 expressed on the surface of cancer cells. J. Immunol..

[30] Antonioli L., Blandizzi C., Malavasi F., Ferrari D., Hasko G. (2016). Anti-CD73 immunotherapy: A viable way to reprogram the tumor microenvironment. Oncoimmunology.

[31] Young A., Ngiow S.F., Barkauskas D.S., Sult E., Hay C., Blake S.J., Huang Q., Liu J., Takeda K., Teng M.W.L. (2016). Co-inhibition of CD73 and A2AR Adenosine Signaling Improves Anti-tumor Immune Responses. Cancer Cell.

[32] Vijayan D., Barkauskas D.S., Stannard K., Sult E., Buonpane R., Takeda K., Teng M.W.L., Sachsenmeier K., Hay C., Smyth M.J. (2017). Selective activation of anti-CD73 mechanisms in control of primary tumors and metastases. Oncoimmunology.

[33] Sharifi M.N., Mowers E.E., Drake L.E., Collier C., Chen H., Zamora M., Mui S., Macleod K.F. (2016). Autophagy Promotes Focal Adhesion Disassembly and Cell Motility of Metastatic Tumor Cells through the Direct Interaction of Paxillin with LC3. Cell Rep..

[34] Shang L., Chen S., Du F., Li S., Zhao L., Wang X. (2011). Nutrient starvation elicits an acute autophagic response mediated by Ulk1 dephosphorylation and its subsequent dissociation from AMPK. Proc. Natl. Acad. Sci. USA.

[35] Jiang C.F., Shi Z.M., Li D.M., Qian Y.C., Ren Y., Bai X.M., Xie Y.X., Wang L., Ge X., Liu W.T. (2018). Estrogen-induced miR-196a elevation promotes tumor growth and metastasis via targeting SPRED1 in breast cancer. Mol. Cancer.

[36] Ji X., Peng Z., Li X., Yan Z., Yang Y., Qiao Z., Liu Y. (2017). Neutralization of TNFalpha in tumor with a novel nanobody potentiates paclitaxel-therapy and inhibits metastasis in breast cancer. Cancer Lett..

